# Thermostable enzyme research advances: a bibliometric analysis

**DOI:** 10.1186/s43141-023-00494-w

**Published:** 2023-03-27

**Authors:** Che Haznie Ayu Che Hussian, Wai Yie Leong

**Affiliations:** INTI International University & Colleges, Nilai, Negeri Sembilan Malaysia

**Keywords:** Industrial enzymes, Bioprocess, Biodegradation, Biofuel, Research trends, Bibliometric, VOSviewer

## Abstract

Thermostable enzymes are enzymes that can withstand elevated temperatures as high as 50 °C without altering their structure or distinctive features. The potential of thermostable enzymes to increase the conversion rate at high temperature has been identified as a key factor in enhancing the efficiency of industrial operations. Performing procedures at higher temperatures with thermostable enzymes minimises the risk of microbial contamination, which is one of the most significant benefits. In addition, it helps reduce substrate viscosity, improve transfer speeds, and increase solubility during reaction operations. Thermostable enzymes offer enormous industrial potential as biocatalysts, especially cellulase and xylanase, which have garnered considerable amount of interest for biodegradation and biofuel applications. As the usage of enzymes becomes more common, a range of performance-enhancing applications are being explored. This article offers a bibliometric evaluation of thermostable enzymes. Scopus databases were searched for scientific articles. The findings indicated that thermostable enzymes are widely employed in biodegradation as well as in biofuel and biomass production. Japan, the United States, China, and India, as along with the institutions affiliated with these nations, stand out as the academically most productive in the field of thermostable enzymes. This study’s analysis exposed a vast number of published papers that demonstrate the industrial potential of thermostable enzymes. These results highlight the significance of thermostable enzyme research for a variety of applications.

## Background

Recent developments in biological based materials for a variety of applications have begun to penetrate the industrial sector [1, 2]. As a result, several industries are now taking steps to transition from chemical-based manufacturing to clean biological manufacturing. [3–5]. Enzymes are proteins that operate as biological catalysts in biological systems, speeding up reactions and catalyzing chemical reaction functions [6–8]. Enzymes are increasingly being used in a wide range of industrial processes due to their great specificity of action. Their benefits include their efficiency in speeding chemical reactions and their selectivity in distinguishing between potential substrates [6, 9, 10]. Enzymes may aid in the development of environmentally friendly processes by displacing toxic chemicals used in industrial manufacturing [11–13].

Enzymes can be utilized in many industrial production processes, enabling the creation of environmentally friendly technology processes without creation of waste and production of hazardous chemicals such as detergent formulations, cheese production, the leather industry and pharmaceuticals industry [8, 14–17]. However, the most major difficulty to the widespread commercial deployment of enzymes is their intrinsic fragility under rigorous industrial operations conditions, one of which is that they cannot sustain the process's high temperature. The majority of enzymes lose activity at higher temperatures, which are typically between 25 °C and 37 °C [18]. Enzymes derived from thermophiles have affected the interest of numerous businesses, including the pharmaceutical, detergent, textile, food, feed industries, leather, and paper, as well as biorefineries [19, 20]. The term for these enzymes is “thermostable enzymes”.

Thermostable enzymes are enzymes that can resist high temperatures, typically between 45 °C and 120 °C [2]. These enzymes not only survive at high temperatures, but they also work in severe environments where humans cannot exist. Thermostable enzymes provide numerous benefits to the industrial sector, including a rapid growth rate, a reduced risk of contamination, a reduction in liquid viscosity, and improved solubility in polymeric substrates and oil [21]. The resistance of thermostable enzymes to proteolysis and chemical denaturation is greater. With these benefits, it is possible to slow down the process of denaturation, which is essential for commercial preparations, and to store them at room temperature for a longer half-life. Many different enzymes have been identified from thermophiles, including cellulases, amylases, xylanases, pectinases, proteases, and lipases [22–25].

Numerous thermophilic microbial taxa, such as *Bacillus*, *Clostridium*, *Pyrococcus*, *Thermus, Thermotoga*, and *Aquifex*, produce unique enzymes including α-amylase, lipase, cellulase, xylanase, alkaline phosphatase, polymerase and ligase [18]. *Taq* polymerase was the first thermostable enzyme to be reported in 1976 [26]. It was discovered from thermal springs of Yellowstone National Park in 1969 and was isolated from *Thermus Aquaticus*. The ideal temperature for activity was determined to be 75–80 °C, with a half-life of 2 h at 92.5 °C, 40 min at 95 °C, and 9 min at 97.5 °C [27]. Formerly, DNA polymerases obtained from *Escherichia coli* were used in polymerase chain reaction (PCR) methods [28]. Nevertheless, they lost their enzymatic activity at high temperatures, necessitating the addition of a new polymerase enzyme after each cycle of denaturation and primer hybridization, which was time-consuming and costly. As a result, the availability of thermostable Taq DNA polymerase has an impact on the PCR development process since it can survive the 95 °C required for DNA strand separation without denaturing. Thermophilic microorganisms are occupying several biological niches, including hot springs, deep marine, volcanic sites, compost and deep organic landfills [29]. However, they have been widely investigated in hot springs around the world and are abundant in nature [30]. Hot springs have been identified as natural habitats that are ideal for thermophile colonization.

Since 1970, numerous studies have been undertaken on thermostable enzymes. As this protein differs from mesophilic enzymes in its structural properties and adaptations to the harsh environment, the majority of the studies have been oriented to studying these characteristics [25, 31]. Thermophiles have rigid cell walls, a high G + C concentration of DNA contents that ultimately alters these protein structure [32]. This component causes these enzymes to have distinct hydrogen bonds, electrostatic interactions, hydrophilic contacts, metal binding and loop deletion or shortening, which eventually results in a superior conformational shape. Enzyme thermostability is typically an intrinsic feature determined by the primary protein structure. The thermostable enzymes were more stable than the mesophilic enzymes because they had larger levels of non-polar amino acids [33]. These amino acids increase the hydrophobicity of proteins, which is directed towards the catalytic pocket and increases the rigidity of proteins [34]. Furthermore, the higher charged of amino acids also enhance the electrostatic interactions in the outer part of protein leading greater ion pair interaction [35]. Studies also revealed that thermostable enzymes contain higher disulphide bonds and hydrophobic bonds [36]. These criteria make the structure of the enzymes more rigid and lead to better folding of the conformational.

Technologies for data extraction and synthesis are currently essential due to the abundance of data available. Bibliometric analysis is a statistical methodology that use [37, 38] to analyze and evaluate a significant number of scientific research articles in various fields of knowledge. Bibliometric is very important for uncovering developing trends in a specific topic or field by identifying the relationship of core research or authors across all publications, journal performance, collaboration patterns, and research constituents [39]. The findings of academic publishing analysis have proven to be an excellent method of measuring the impact of research trends [40]. As a result, the researcher can find knowledge gaps concerning the issue and develop new original ideas for investigation and contribution to the specific research topic. Through bibliometric analysis, it is possible to gain a comprehensive understanding of a certain topic and its relationship to specific databases. Utilizing interaction charts, this popular method provides a simple and easy assessment of selected works. The publication year, the most-cited articles, journals, authors, and fields of study were all examined in this study's bibliometric analysis of thermostable enzymes. Current trends in thermostable enzymes are examined in light of relevant research.

### Scientific literature research

**Fig. 1:**
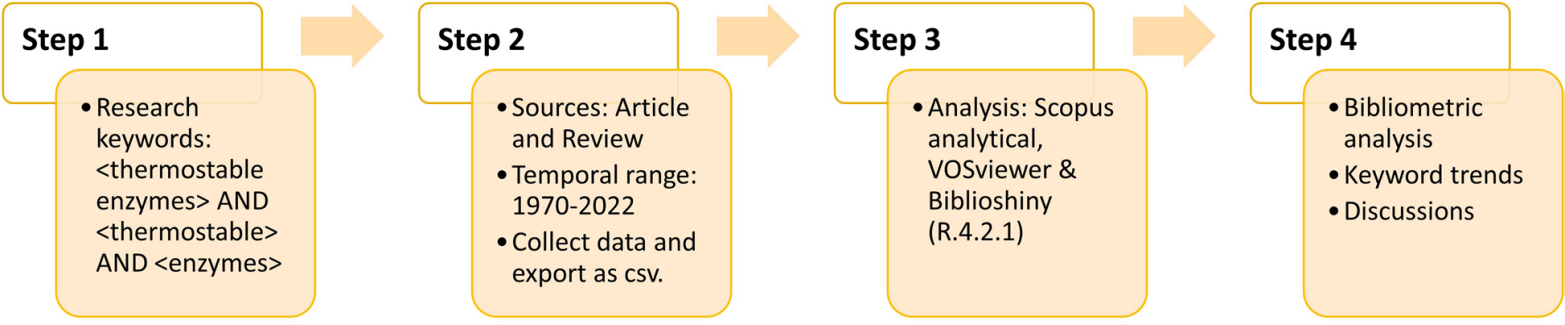
Flowchart of bibliometric search strategy

The Scopus database (www.scopus.com) was recovered in September 2022 using the search terms "thermostable enzyme" AND "thermostable" AND "enzymes" (Fig. 1), with only research publications and review studies included. There were no time restrictions placed on the search, and all publications up to the search year (1970–2022). This allows us to estimate the global research output on thermostable enzymes from a large number of high-quality journal papers. The "analyze results" feature of Scopus's search tools displays the publication year and topic matter of the chosen work. The articles we gathered were examined using VOSviewer version 1.6.15, which was used to import all data into a Microsoft Excel spreadsheet (csv) (www.vosviewer.com). VOSviewer is a software tool used in bibliometric analysis by constructing and visualising the networks of publications, documents, sources, authors, organizations, and countries. These networks can be built via citation, bibliographic coupling, co-citation, or co-authorship relationships. It also provides network mapping of the relationships between bibliometric networks and the topic of interest. In this study, the data were collected to determine the most productive countries, the most highly referenced journals, authors, publications and research trends based on keyword analysis. Several different types and units of analysis were used to construct the results.

### Analysis of publications

#### General analysis

**Fig. 2:**
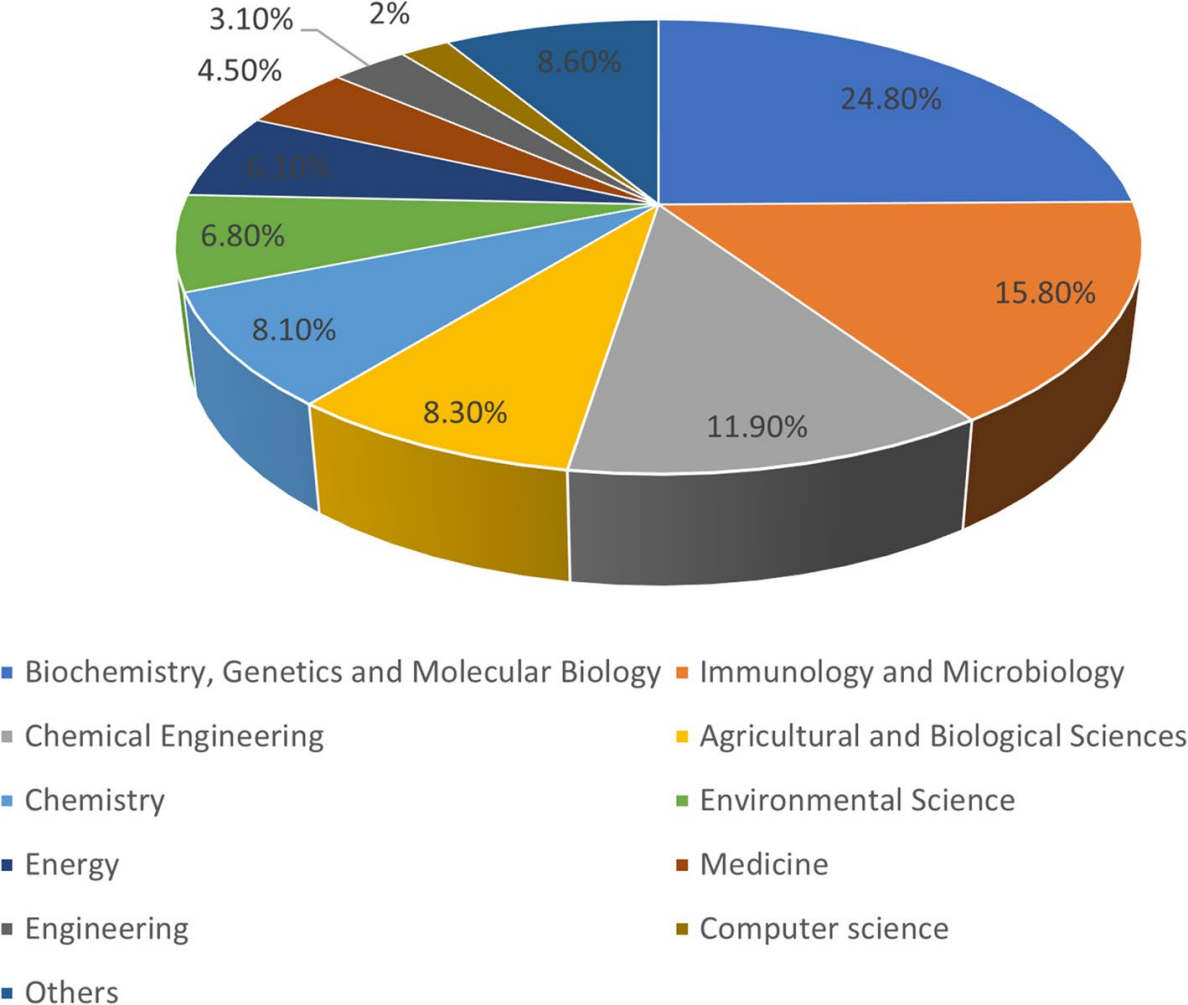
The main research topic related to thermostable enzymes (Scopus)

**Fig. 3:**
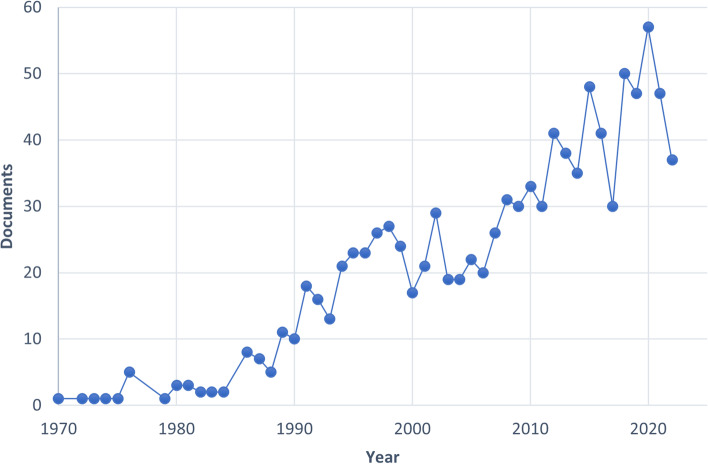
Annual scientific production of articles on thermostable enzymes

The aforementioned search method accumulates 1090 articles, which were divided into 11 categories of research topics (Fig. 2). The search for articles and reviews was narrowed down to 1010 publications. 97% (*n* = 3925) of them were written in English, 1.03% (*n* = 42) in Chinese and 1.97% in other languages. The first study on thermostable enzymes published was the purification of thermostable isoleucyl-tRNA synthetase from *Bacillus stearothermophilus* in 1972 [41]. After 1995, the number of articles increased each year, finally surpassing 20 and going over 40. The distribution of articles by study area from 1970 to 2022 is shown in Fig. 3. Qualitative trend analysis for each study area with more than 100 publications between 2017 and 2022 was conducted to examine the most common use of thermostable enzymes during the preceding five years (biochemistry, genetic and molecular biology, immunology and microbiology, chemical engineering, chemistry and agricultural and biological sciences).

##### Biochemistry, genetics and molecular biology

Study on the isolation and characterization [42–45], expression and purification [46–50], gene cloning, structural function analysis, improving catalytic efficiency, site-directed mutagenesis, protein crystallization, computational simulations, rational engineering to improve enzymes activity [51–56] preservation of enzymes, indigenous thermophilic exploration, cell-free enzymatic, polymerase synthesis, marine thermophiles, enzymatic purification and biodegradation [57–59]. The most important enzymes: Cellulose, xylanase, lipase and amylase.

##### Immunology and microbiology

Study on immunogenicity of subunit vaccine [60], biosensor, detection of organic pollutants [61], enzymatic bioreceptor [62], applications to in vitro biosynthesis, substrate specificity, improvement catalytic performance, biomedical, preparation of pharmacologically active icaritin, in vitro antioxidant activity, cancer prodrug-mediated therapies or gene therapy applications [63–65]. The most important enzymes: Esterase-2, endoglucanase, aldehyde dehydrogenase, cellulose, xylanase and laccase.

##### Chemical engineering

Study on dye-linked L-lactate dehydrogenase [66], lignification [67], dishwashing machine [68], degradation of lignocellulose [69], biodegradable polymer, biotransformation, fine chemical industry, bio-bleaching and dye decolorizing agent [70], renewable bioethanol [71] and enzyme immobilization for the hydrolysis reaction [72]. The most important enzymes: Lipase, cellulase, xylanase, glucosidase and amylase.

##### Agricultural and biological sciences

Study on class III peroxidases (POX) plants [73], Calotropis procera root peroxidase (CPrP) [74], oxidoreductive enzymes, microalgal and cyanobacterial [75] and bioremediate phenol from petroleum effluent [57]. The most important enzymes: Amylase, peroxidase, esterase.

##### Chemistry

Enzymes-based sensor [76], Flavoenzyme dye-linked L-lactate dehydrogenase (Dye-LDH) [66], degradation of poly (lactic acid), PLA, biodegradation of xenobiotics [77], aromatic compounds and lactic acid, enzyme immobilization on carboxymethyl cellulose (CMC)-hydrogel, organic chemistry, synthetic catalyst and bioremediation—dimethylformamidase (DMFase) [78]. The most important enzymes: Dehydrogenase, peroxidases, phosphatase and pectate lyse.

### Research trends

According to the results of a general analysis, most research on thermostable enzymes has been carried out in the fields of biochemistry, genetics and molecular biology [79, 80]. All studies on this topic, were focuses more on strategies used to enhance thermostable enzymes, such as molecular, characterization, genetic alteration to improve catalytic activity and purification. Although the quest for thermophiles began 40 years ago, the discovery of important and novel thermostable enzymes continues to rise, making the search for and isolation of thermophiles an essential topic of research. However, based on the industrial potential of thermostable enzymes, the majority of research focuses on lignocellulosic biodegradation for biofuel production. The biodegradable process requires severe conditions for the hydrolysis of lignocellulosic biomass [67, 79]. Enzymatic degradation is the most effective and environmentally safe method for converting complex lignocellulose polymers into fermentable monosaccharides, compared to chemical and physical procedures. Cellulase and xylanase are the thermostable enzymes that are involved in this industrial sector [4, 5, 12]. The usefulness of thermostable cellulases and xylanases is primarily determined by their productivity, thermostability, specific activity, broad pH range and broad substrate specificity. Using genetic engineering, expression control and enzyme immobilization, the thermostability of thermophile cellulase and xylanase has been increased in order to expand their industrial applications. Most approaches involve site-directed mutagenesis, whereas cloning was used to increase the enzymes' stability.

### Top research institutions and countries

**Fig. 4:**
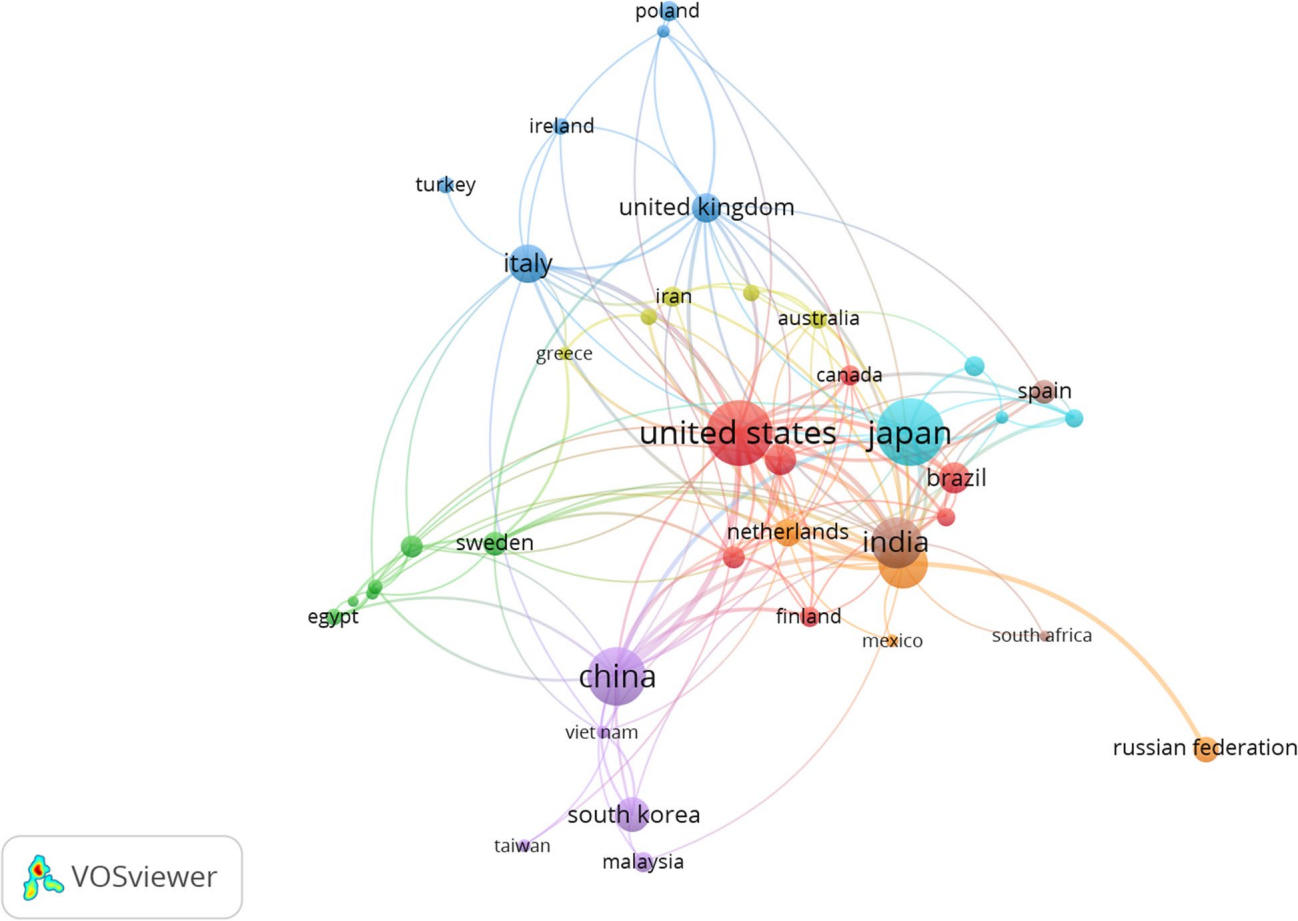
Collaborative networks between the 20 most productive countries in the research of thermostable enzymes according to a bibliometric analysis of the Scopus database

**Fig. 5:**
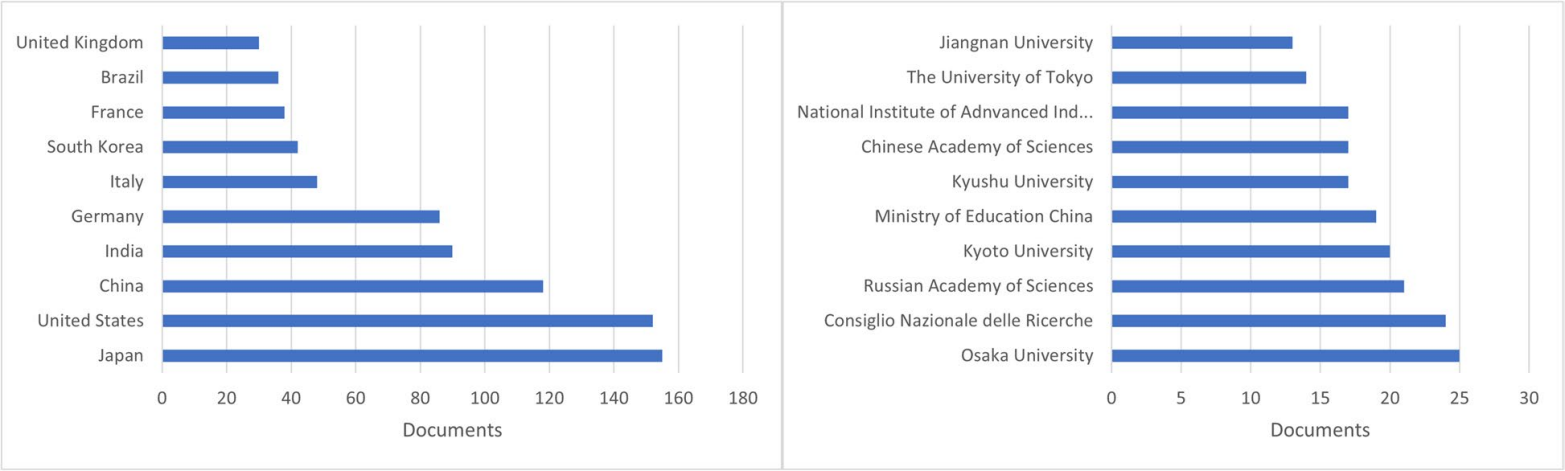
Number of publications per country and top research institutions on thermostable enzyme research based on the Scopus database

A review of publications by nation revealed that the top 10 countries represented 79.90% (*n* = 807) of all articles (Fig. 4). Japan came in first with 15.6% of all papers produced, followed by the United States (15%), China (11.88%) and India (9.10%). The top 10 institutions with the most publications included three Chinese universities and five Japanese universities. Other countries, such as India, Germany, South Korea and the United States (US), did not have any institutions in the top 10, while the US had just one. From search results analysis based on document affiliations, Osaka University Japan was the top research institution for the study of thermostable enzymes with 25 documents followed by the Consiglio Nazionale delle Ricerche Italy, the Russian Academy of Sciences, Kyoto University and the Ministry of Education, China (Fig. 5).

### Most global cited documents

We examined the number of citations of articles published between 1976 and 2022 to identify the most cited articles of recent times and determined that highly referred publications are often older. As indicated in Table 1, Holland (1991) [81] had the most referenced articles throughout this time span, with 2,147 citations. The 5'-3' exonuclease activity of Taq DNA polymerase from *Thermus aquaticus* was used by the authors to create a simple and effective approach for identifying PCR products. This enzyme is frequently employed for PCR amplification because of its exceptional heat resistance. At 72 °C, nucleotides are integrated at a rate of 2 and 4 kb/min, while their half-life at 95 °C is 40 min.

**Table 1:** Top 10 most cited publications on thermostable lipase in the Scopus database (1970–2022)

Author	Year	Journal	Title	Total Citation	Citation
Holland PM, Abramson RD, Watson R, and David Gelfand H	1991	Proc Natl Acad Sci U S A	Detection of specific polymerase chain reaction product by utilizing the 5' -* 3' exonuclease activity of *Thermus aquaticus* DNA polymerase	2147	[81]
Haki GD and Rakshit SK	2003	Bioresour Technol	Developments in industrially important thermostable enzymes: a review	885	[82]
Barany, F	1991	Proc Natl Acad Sci U S A	Genetic disease detection and DNA amplification using cloned thermostable ligase	647	[83]
Fernandez-Lafuente, R	2010	J Mol Catal B Enzym	Lipase from Thermomyces lanuginosus: Uses and prospects as an industrial biocatalyst	449	[84]
Klibanov AM	1983	Adv Appl Microbiol	Stabilization of Enzymes against Thermal Inactivation	406	[85]
Turner P, Mamo G and Karlsson EN	2007	Microb Cell Fact	Potential and utilization of thermophiles and thermostable enzymes in biorefining	386	[86]
Sterner R and Liebl W	2001	Crit Rev Biochem Mol Biol	Thermophilic Adaptation of Proteins	318	[87]
Zhulidov PA, Bogdanova EA, Shcheglov AS, Vagner LL, Khaspekov GL, Kozhemyako VB, Matz MV, Meleshkevitch E, Moroz LL, Lukyanov SA, Shagin DA	2004	Nucleic Acids Res	Simple cDNA normalization using kamchatka crab duplex-speci®c nuclease	316	[88]
Berka RM, Grigoriev et al	2011	Nat Biotechnol	Comparative genomic analysis of the thermophilic biomass-degrading fungi Myceliophthora thermophila and Thielavia terrestris	309	[89]
Eom S, Wang J, Steitz T	1996	Nature	Structure of Taq polymerase with DNA polymerase active site	305	[90]

The second most cited review article, ‘Developments in Industrially Significant Thermostable Enzymes: A Review ‘ by Haki and Rakshit [82], with 883 citations. The authors are connected to the Thai Asian Institute of Technology's Bioprocess Technology Program (AIT). The number of applications for enzymes has increased as a result of the creation of thermostable enzymes, as this review article explains. Due to their inherent stability, thermophilic organisms have discovered a variety of economic applications as a result of the numerous studies that have been conducted to identify them. The food industry (which synthesizes amino acids), the petroleum, chemical and paper sectors are the next largest users of thermostable enzymes in the starch sector [91–93].

Barany is the author of third most cited article which published in the same journal as Holland 1991, *Journal of Proceeding Natl Acad Sci USA*. Barany is a researcher from the Cornell University Medical College, New York has conducted a study on genetic disease detection and DNA amplification using cloned thermostable ligase from *Thermus aquaticus* [83].

Fernandez-Lafuente is the fourth most cited review article with total citation about 449. Fernandez-Lafuente is a researcher from the Instituto de Catálisis-CSIC, Spain. The most cited article in 2010 is specific for thermostable lipase from *Thermomyces laguginosus* which available in both soluble and immobilized form [84].

### Most relevant authors

Rossi Mosè E, a researcher at the Consiglio Nazionale delle Ricerche in Rome, Italy, is the most cited and notable author. His 445 papers were cited 11,991 times in 6737 different documents. The author has written 13 articles on the study of thermostable enzymes, and the paper titled "Crystal structure of the most catalytically effective carbonic anhydrase enzyme, SazCA from the thermophilic bacterium *Sulfurihydrogenibium azorense*" has received the most citations, with 62 citations [52]. Oh Deokkun, a scholar at Konkuk University in Seoul, South Korea, is the second most cited author (8286 citations). The most recent publication, which has 74 citations, was released in 2011 where the research was conducted on cloning and expression of thermostable cellobiose 2-epimerase, a from *Caldicellulosiruptor saccharolyticus* into Escherichia coli as expression host [44].

**Fig. 6:**
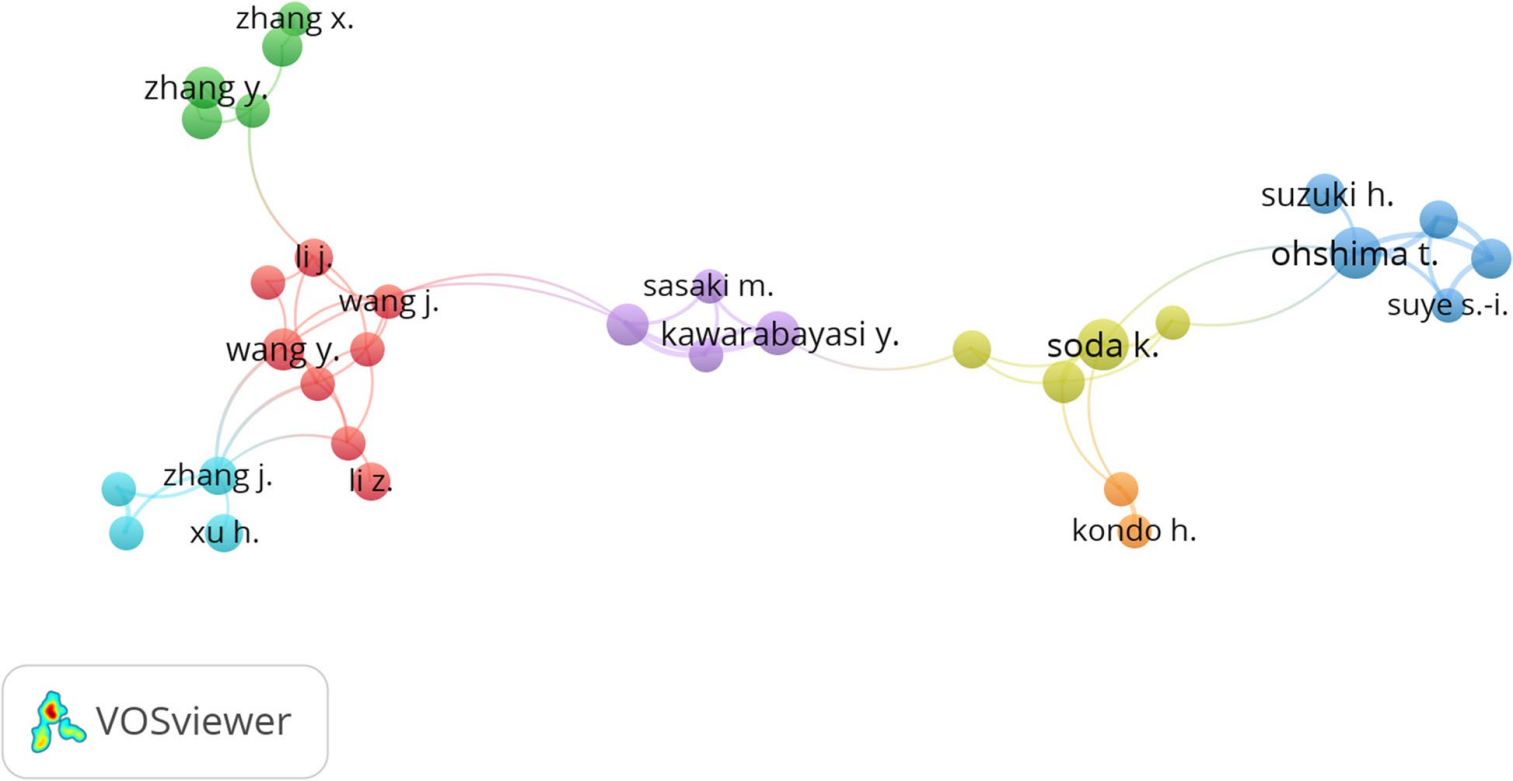
The top 30 most productive authors collaborate in thermostable lipase research according to a bibliometric analysis of the Scopus database

The collaborative network among authors has been analyzed. From the VOSviewer bibliometric analysis in Fig. 6, there are seven clusters of authors that connected with each other on thermostable enzyme research, but Rossi Mosè and Oh Deokkun were not included in the collaborative network. However, Ohshima, T (Cluster 3) is the third most cited author (3315 citations) and a researcher from Osaka Institute of Technology, Japan. He has connections with the fourth most cited author, Soda. K (Cluster 4) who is from the Institute of Chemical Research, Kyoto University. Both of them published a review paper together in 1989 with the title’Thermostable amino acid dehydrogenases: applications and gene cloning ‘ which was cited by 33 authors [94]. Cluster 1 consists of eight connected authors, which are Gao, R., Wang, Y., Wang, Xiaojuan, Wang, Zhongyu (Jilin University, China). They are members of the same research group at Key Laboratory for Molecular Enzymology and Engineering.

### Recent research

Recent research on thermostable lipase was analyzed in the Scopus database from 2018–2022 (5 years). From the results, Febbraio Ferdinando from Consiglio Nazionale delle Ricerche, Rome, Italy (same affiliations with the most relevant authors, Rossi Mosè) have much more research on enzyme-based biosensor by using thermostable lipase. The most recent research paper on biosensor fluorescent detection of organophosphate pesticides using the thermostable enzyme esterase-2 from *Alicyclobacillus acidocaldarius* (EST2) with a lipase-like Ser-His-Asp catalytic triad was published in 2022, and is a promising candidate as a bioreceptor for the development of biosensor [62].

Huiying Luo, a researcher at the Chinese Academy of Agricultural Sciences in Beijing, China, studies thermostable xylanase and cellulase, which are commonly used to decompose lignocellulosic biomass and have potential applications in the feed and fuel industries [95, 96]. This study sheds light on the underlying mechanism and methods of modifying xylanase for commercial use.

### Most relevant journals

The *Journal of Applied Microbiology and Biotechnology*, published by Springer Nature, ranked first, with 43 papers and 1066 citations. Table 2 shows that 20% of all publications on the subject may be attributed to the top 10 journals. From 43 documents, the highest cited paper is written by Bragger (1989) with research on extremely thermophilic archaebacteria and eubacteria with 101 citations [97]. This article demonstrated the isolation of 36 thermophilic eubacteria for extracellular amylase, hemicellulase (xylanase), cellulase, protease, pectinase and lipase activities. As shown in Table 3, the journal had an impact factor of 3.3 and a CiteScore of 8.8 in 2019; thus, it received an average of 8.8 citations per article published. This publication is of tremendous relevance in the field since, as its title suggests, it focuses primarily on the application of microorganism-derived enzymes in biotechnology.

**Table 2:** Top 10 of the most relevant sources for thermostable enzymes research

Journal	Country	Articles No	Publisher	Impact factor	Cite score	H-index
Applied Microbiology and Biotechnology	Germany	43	Springer Verlag	3.3	8.8	236
Enzyme And Microbial Technology	United States	36	Elsevier	3.705	6.0	153
Bioscience, Biotechnology and Biochemistry	United Kingdom	18	Oxford University Press	2.337	3.3	123
Biotechnology Letters	Netherlands	17	Springer Nature	2.716	4.0	114
Applied Biochemistry and Biotechnology	United States	16	American Society for Microbiology		7.8	339
Journal Of Biochemistry	United Kingdom	16	Oxford University Press	3.241	4.5	120
Applied And Environmental Microbiology	United states	15	American Society for Microbiology	2.926	7.8	339
Biotechnology for Biofuels	United Kingdom	15	Biomed Central Ltd	7.670	11.5	108
International Journal of Biological Macromolecules	Netherlands	15	Elsevier	8.025	11.6	144
Bioresource Technology	United Kingdom	13	Elsevier	11.889	17.4	317

**Table 3:** Keyword clusters analysis of scientific publication of thermostable enzymes in the Scopus collection database (1976–2022)

Cluster	Items
1	Amylase, bacillus, bacteria, bacterial enzymes, bacterial strains, bacterium, beta glucosidase, biocatalyst, biofuel, biomass, biosynthesis, biotechnology, cellulase, cellulose, enzyme assay, enzyme immobilization, enzyme synthesis, fermentation, fungi, genetic engineering, geobacillus, geobacillus stearotherm, glucose, hydrolysis, microbiology, phylogeny, starch, thermodynamic stability, thermophile, thermophilic bacteria, thermostability, thermostable enzyme, triacylglycerol lipase, xylan endo 1,3 beta xyloses
2	Bacterial protein, biocatalysis, catalysis, catalytic domain, chemistry, crystal structure, x-ray crystallography, enzyme active site, enzyme structure, enzymology, glycosidase, glycoside hydrolases, mutagenesis, protein confirmation, protein denaturation, protein engineering, protein stability
3	Amino acid, archeal proteins, cloning, molecular DNA, Escherichia coli, gene expression, gene sequence, hydrogen-ion concentration, isolation and purification, molecular cloning, molecular genetics, nucleotide sequences, polymerase chain reaction, protein expression, protein purification, recombinant protein, sequence alignment, sequence homology, thermotoga maritima, thermus, thermus thermophilus
4	Bacterial enzymes, enzyme analysis, enzymes inhibition, enzymes kinetics, enzyme purification, enzyme specificity, enzyme substrate, substrate specificity, substrates
5	Thermotoga maritama

The *Journal of Enzyme and Microbial Technology*, with 36 articles, is the second most relevant publication in the field. Its publications had to do with technological advancements. *Bioresource Technology* is the most prominent journal on the subject, with an impact factor of 11.889 and 13 articles. This journal published Haki & Rakshit (2003), which is one of the top 10 most referenced papers with 885 citations. Their principal fields of publication were Bioscience, Biotechnology, and Biochemistry ranked third among the most cited journals with twenty citations for 18 published publications. 33% of the top ten journals were published in the United States, 30% in the United Kingdom, 21% in Germany, and 16% in the Netherlands. Thus, 67% of the journals were European and 33% were North American.

### Keyword trends analysis

**Fig. 7:**
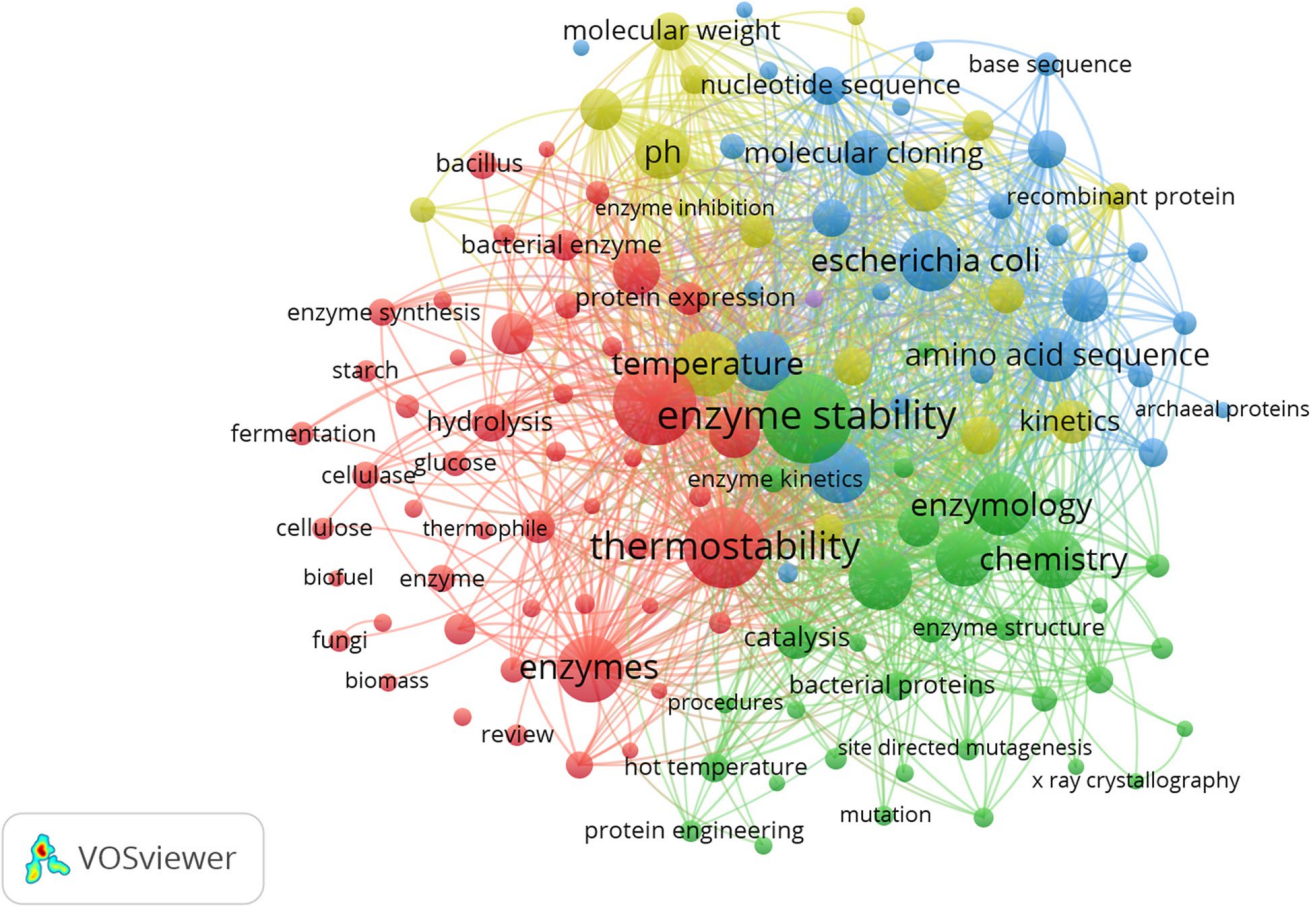
Keyword trend analysis of scientific publications on thermostable enzymes in the Scopus collections database (1976-2022)

The purpose of keyword co-occurrence analysis is to identify emerging trends and hot subjects, and it is an important method for tracking scientific progress. The findings of the trend analysis for the time periods utilising keywords with at least 30 occurrences are displayed in Fig. 7 and Table 3. The result revealed that there were 7996 keywords in the 1,010 articles, and 135 keywords appeared 30 times or more. For better interpretation of results, the terms used for the literature search were omitted from Fig. 7. From the results, 5 clusters were obtained from total keywords. Analysis of the keyword trend revealed that studies were associated with amylase, beta glucosidase, biofuel, cellulase, and cellulose are included in cluster 1 (Table 3). We highlight the fact that research on thermostable enzymes and biofuel production began to emerge strongly in this time range.

## Conclusions

This study has detailed the current research status on thermostable enzymes that has increased throughout the years. We detected a trend toward the application of thermostable enzymes in several industrial research domains, notably for ecologically friendly approaches to address pollution and bioremediation. Enzymatic production of biodiesel is expected to be a trend in the coming years, encouraged by the increasing interest in natural components and green technologies. The usage of thermostable enzymes in industrial applications is expected to increase especially in biodegradable of lignocellulosic biomass for biofuel production. However, we noticed weak collaboration links between researchers from different nations, and organizations which have to be developed to increase knowledge diffusion. There has been an increasing amount of study and Japan remains ahead in both the sum of publications and total citation frequency in this sector. Thus, it is not difficult to forecast that this area of research is expected to continue to rapidly increase and that more papers will be published in the coming years. For future research, it will be necessary to create strategies for developing thermostable enzymes that can be employed extensively in the biofuel, biodegradation, food, pharmaceutical, textile, bio-based, and animal feed industries. Enzymes are frequently denatured by high temperatures, strong acids and bases, organic solvents, and other harsh conditions, compromising their catalytic capabilities and limiting their applicability in industrial processes. Discovering new thermostable enzymes in extreme environments or performing molecular modification of existing enzymes with poor thermostability using emerging protein engineering technology are now effective methods for getting new thermostable enzymes.

## Data Availability

All research manuscripts and review data utilised in this study were retrieved in CSV format from the Scopus database (attached in supplementary materials). This link yielded all of the evaluated search results.https://www.scopus.com/term/analyzer.uri?sid=4e9621c056d6ac82d181afb073c5e641&origin=resultslist&src=s&s=TITLE-ABS-KEY%28thermostable-enzymes%2c+thermostable%2c+enzymes%29&sort=plf-f&sdt=cl&sot=b&sl=58&count=1022&analyzeResults=Analyze+results&cluster=scosubtype%2c%22ar%22%2ct%2c%22re%22%2ct%2bscopubstage%2c%22aip%22%2cf%2bscosrctype%2c%22d%22%2cf&txGid=12bdad479e4e1e91daabb8fc725d2e54

## Abbreviations

CSV: Comma Separated Value (CSV)
RNA: Ribonucleic acid
DNA: Deoxyribonucleic acid
PCR: Polymerase chain reaction
POX: Peroxidases
CPrP: Calotropis procera root peroxidase
LDH: L-lactate dehydrogenase
PLA: Poly lactic acid
CMC: Carboxymethyl cellulose
CA: Carnonic anhydrase
DMFase: Dimethylformamidase
